# QTL Mapping of Seed Vigor of Backcross Inbred Lines Derived From *Oryza longistaminata* Under Artificial Aging

**DOI:** 10.3389/fpls.2018.01909

**Published:** 2018-12-21

**Authors:** Jie Jin, Weixiong Long, Liuting Wang, Xingdan Liu, Guojing Pan, Wei Xiang, Nengwu Li, Shaoqing Li

**Affiliations:** ^1^State Key Laboratory of Hybrid Rice, Key Laboratory for Research and Utilization of Heterosis in Indica Rice of Ministry of Agriculture, Engineering Research Center for Plant Biotechnology and Germplasm Utilization of Ministry of Education, College of Life Sciences, Wuhan University, Wuhan, China; ^2^College of Agronomy, Hunan Agricultural University, Changsha, China

**Keywords:** seed vigor, QTLs, *O. longistaminata*, wild rice, artificial aging

## Abstract

Seed vigor is an important character of seed quality that promotes rice to germinate rapidly from soil and developing to a strong seedling, especially in the current rice direct-sowing production system. However, previous studies for seed vigor mainly concentrate in cultivars, and less reports involving in wild rice. In this study, 152 backcross inbred lines (BILs) derived from wild rice *Oryza longistaminata* were genotyped with re-sequencing technology, and QTLs for seed vigor related traits under normal and artificial aging treatment were analyzed. Totally, 36 QTLs were detected, of which, eight for germination potential (GP), 10 for germination rate (GR), 9 for seedling length (SL), and 9 for root length (RL). Among these, 14 novel QTLs were identified from *O. longistaminata*. Of which, six QTLs were related to germination, and eight related to seedling growth under aging stress. What’s more, the major QTLs *q9SL1.1*, *q6SL1.1*, and *q3SL1.1* for seedling length were fallen in the same locus and fine-mapped an interval about 90 Kb. The major QTLs *q9GR8.1* and *q9GP8.1* related with germination were fine-mapped to an interval about 90 Kb. This work will provide us basis for breeding of high seed vigor rice in rice breeding programs and further cloning of these genes.

## Introduction

As rapid development of economy, rice production is transferring from traditional getting-high-yield to getting-high-efficiency by farmers. Thus, new planting methods like direct seeding is increasingly popularizing in China dues to its lower cost and easy operation (Fujino et al., 2004). High seed vigor, including rapid germination, rapid seeding growth, and good performance after long storage play an important role in direct seeding of rice (Mahender et al., 2015). However, long storage would decrease generation ability of rice grains, hence reduce the seedling vigor, and increase cost of rice production and seed management (Zeng et al., 2006). Especially in southern China, the loss of rice due to seed aging or deterioration under wet and warm weather during rice mature season is becoming a serious problem. Therefore, it is urgent to breed elite rice adapting to long time storage to keep high seed vigor.

Normally, seed vigor assessment will take years under natural storage. Alternatively, a so-called accelerated aging or controlled deterioration test by aging seeds rapidly at elevated temperature and humidity have been developed to mimic natural aging, which will greatly reduce the testing time (Schwember and Bradford, 2010). QTL mapping for seed vigor under artificial aging treatments had been done in 2014 (Han et al., 2014; Ku et al., 2014). Seed vigor is a complex trait which can be influenced by many factors such as seed size, genotype, seed development, and storage time and environment (Sun et al., 2007; Milosevic et al., 2010). Although storage environment are important for seed vigor, genetic factors usually play a vital role for seed vigor (Hang et al., 2015; Sasaki et al., 2015; Dong et al., 2017). In rice, several QTLs for seed germination activity, seedling rate, growth potential including shoot and root length have been detected and mapped (Zhang et al., 2005, 2017; Li et al., 2012; Dargahi et al., 2014; Hang et al., 2015). However, QTLs from wild rice to improve seed vigor have never been reported.

The perennial wild species, *Oryza longistaminata* as the old progenitor both of *Oryza glabberrima* and *Oryza sativa* is believed to be an important potential source with favorable alleles for abiotic tolerance traits (Khush, 1997). In this study, we conducted QTLs analysis of potential seed vigor in *O. longistaminata* by measurement of seed germination rate, germination potential including seedling shoot length and root length, using an advanced BIL population derived from a cross between 9311 and *O. longistaminata*. Totally, 36 QTLs including eight for germination potential, ten for germination rate, and eighteen for growth potential were detected. These QTLs can provide a better understanding of the genetic basis for the seed vigor, and establish the foundation for applying these potential alleles in molecular breeding programs.

## Materials and Methods

### Plant Materials

A set of rice BC_2_F_20_ BILs, derived from a cross between wild rice *O. longistaminata* and elite indica cultivar 9311 as recurrent parent were used for genotyping and phenotyping. All plants were planted during summer, in Ezhou Hubei, China. When the plants matured, the seeds were harvested for experiments in greenhouse.

### Artificial Aging Treatment

Artificial accelerated aging treatment was based on an improved method originally proposed by Zeng et al. (2002). Seeds of the BILs and 9311 were harvested about 40 days after heading at the Hybrid Rice Experimental Base of Wuhan University in Ezhou, Hubei. All the seeds from the 152 BILs and 9311 were treated using a thermostatic moisture regulation at 45°C and 90% humidity for 3, 6, and 9 days, respectively. The untreated seeds were used as control. All treatments followed a complete randomized block design with three replications.

### Correlation Analysis

Correlation was calculated based on two sample *t*-test with equal variances under R package. The R corrplot package^1^ was installed and then loaded to analyze the correlation coefficient between two variables. Correlation among traits was computed at *P* < 0.05 and *P* < 0.01, respectively.

### Germination Assessment and Evaluation of Seedling Vigor-Related Traits

The germination experiment followed a randomized complete block design with three replications and was conducted at 37°C in a growth chamber in 2014. Fifty filled and healthy seeds of the 9311 and each BILs were selected to ensure good sowing quality. The seeds were incubated in a growth chamber at 28°C, 65% relative humidity, illumination conditions of 4000 lux, and a 14/10 (day/night) photoperiod with three replications. The number of germinated seeds was counted at 4th and 10th day. Germination rate data was collected over a 10-day period after sowing and was used for QTL analysis. Seedling root length (RL) and shoot length (SL) investigation were carried out using a hydroponic rice seedling culture model system (Kim et al., 2005), and they were measured at the 10th day after transfer to the hydroponic box, each BILs were randomly selected five plants for assessment. The germination rate (GR) was calculated as GR = n/N × 100%, where n is the total number of germinated seeds at 10th day and N is the total number of seeds tested. The germination potential (GP) was calculated as the number of germinated seeds on 4th day divided by the total number of seeds.

### QTL Analysis

Young-leaves were collected from seedlings of the BILs population, and genomic DNA was extracted using the CTAB method (Healey et al., 2014) with minor modifications. The SNP calling and bin map constructed method were based on the previous report (Huang et al., 2009). Genotypes were called based on SNP ratios. The breakpoints were determined at the boundary of the different genotypes. Bin marker was obtained by combining genotypes with recombination breakpoints. All the resequencing data of BILs combined with parents and the following linkage maps data were not shown. To explore the gene expression for seed germination for the BIL population, QTL IciMapping v3.3 software was used for QTL analysis, threshold LOD of 2.5 was applied (Meng et al., 2015).

## Results

### Seed Germination and Seedling Growth of 9311 and BILs Under Artificial Aging Treatments

**FIGURE 1:**
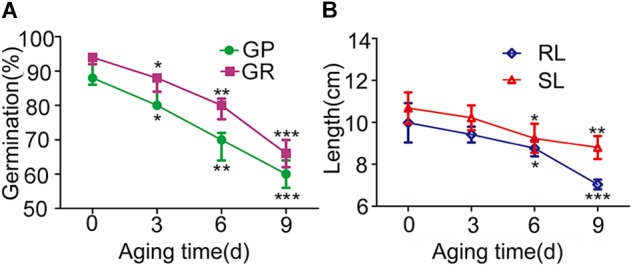
The seed vigor related traits of parent 9311 under artificial aging treatments. **(A)** The blue line with circle indicate germination potential (GP) with the accumulation of artificial aging treatment. The purple line with rectangle show germination rate (GR) under different artificial aging treatments. **(B)** The green line with rhombus indicate seedling length (SL) with the accumulation of artificial aging treatment. The orange line with triangle represent root length (RL) under various artificial aging times. We have compared the traits under artificial treatments with aging time 0 day. Two-way ANOVA method was used to calculate the *P*-value. All the abbreviations below represent the same meaning.^∗^*p* < 0.05; ^∗∗^*p* < 0.01; ^∗∗∗^*p* < 0.001.

To investigate the seed vigor of parent 9311 and *O. longistaminata* BILs and determining appropriate evaluation period of artificial aging in BILs, a time response experiment was firstly conducted using the recurrent parent 9311. Relative to normal conditions, the seed germination capability and seedling growth ability of 9311 gradually decreased as artificial aging treatment prolonged, extremely reduced after 6 and 9 days aging treatments (Figure 1). This indicates that the 6 and 9 days of aging treatment are appropriate to evaluate seed vigor in BILs.

**FIGURE 2:**
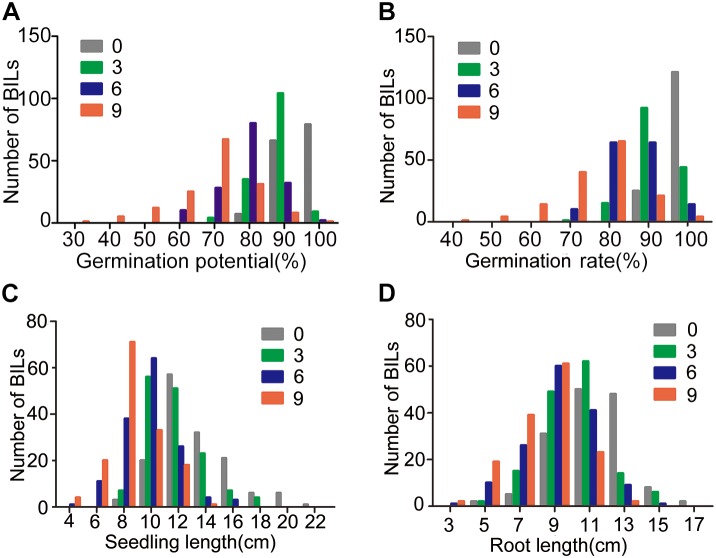
Phenotyping of seed vigor traits of 152 BILs under artificial aging treatments. **(A–D)** represent distribution graph for GP, GR, SL, and RL, respectively.

Similarly, the BIL populations showed a trend in GP, GR, SL, and RL as that of 9311 in response to artificial aging treatments and all the four traits of the BIL population showed continuous distribution patterns under normal growth or artificial aging treatments (Figure 2 and Supplementary Figure S1). The variation range became larger along with elongation of the artificial aging treatment. Most BILs more or less showed a different performance compared with parent 9311 (Supplementary Figure S2).

At the third day of aging treatment, the variation of GP, GR, SL, and RL of BIL population ranged among 0.69∼0.94, 0.72∼0.98, 6.54∼17.71, and 3.87∼14.60 cm, respectively (Figure 2). Correspondingly, the GP, GR, SL, and RL decreased about 0.76∼21.42, 0.70∼26.76, 0.32∼35.75, and 1.97∼36.42%, respectively, relative to the normal growth (Table 1).

**Table 1:** Performance of seed vigor of 9311 and *Oryza longistaminata* BILs under artificial treatments.

Treatment time	Traits	9311	BIL			
			mean	*SD*	min	max
Control (0 day)	GP (%)	0.89 ± 0.03	0.89	0.05	0.73	1.00
	GR (%)	0.93 ± 0.01	0.94	0.04	0.76	1.00
	SL (cm)	9.98 ± 0.94	12.51	2.56	7.84	20.28
	RL (cm)	10.68 ± 1.1	10.50	2.04	4.26	16.34
3 day	GP (%)	0.81 ± 0.02	0.83	0.05	0.69	0.94
	GR (%)	0.87 ± 0.02	0.87	0.05	0.72	0.98
	SL (cm)	9.22 ± 0.55	10.27	2.09	6.54	17.71
	RL (cm)	10.36 ± 0.53	9.20	1.39	3.87	14.60
6 day	GP (%)	0.69 ± 0.04	0.74	0.07	0.51	0.92
	GR (%)	0.79 ± 0.03	0.80	0.07	0.61	0.95
	SL (cm)	8.96 ± 0.34	8.76	2.08	3.26	15.84
	RL (cm)	9.24 ± 0.7	8.22	2.00	2.69	13.55
9 day	GP (%)	0.6 ± 0.04	0.64	0.11	0.22	0.91
	GR (%)	0.66 ± 0.04	0.71	0.10	0.32	0.93
	SL (cm)	8.16 ± 0.38	7.60	1.88	2.07	12.77
	RL (cm)	8.6 ± 0.39	7.33	1.77	2.29	12.07


At the sixth day of aging treatment, the variation of GP, GR, SL, and RL of BIL population ranged among 0.51∼0.92, 0.61∼0.95, 3.26∼15.84, and 2.69∼13.55 cm, respectively (Figure 2). Correspondingly, the GP, GR, SL, and RL decreased about 3.05∼42.36, 2.10∼36.36, 6.31∼59.58, and 5.08∼47.52%, respectively, relative to the normal growth (Table 1).

At the ninth day treatment, the variation of GP, GR, SL, and RL of BIL population ranged among 0.22∼0.91, 0.32∼0.93, 2.07∼12.77, and 2.29∼12.07 cm, respectively (Figure 2). Correspondingly, the GP, GR, SL, and RL decreased about 5.00∼71.05, 5.59∼66.20, 7.22∼78.50, and 5.42∼72.89%, respectively, relative to the normal growth (Table 1). These results indicate that the seed vigor of *O. longsitaminata* BILs showed great variation among different BIL lines, and SL and RL were more sensitive than the GP and GR to the aging treatment.

Correlation analysis between seed vigor related traits was performed by regressing phenotypic values of one trait on those of the others. GP values showed significantly positive correlation with GR values both in normal condition and artificial aging treatment condition. SL values showed positive correlation with RL, but both of SL and RL pose a negative correlation with GR and GP in normal growth, and artificial treatment. What’s more, the correlation of the same seed vigor trait among different treatments was significantly positive (Supplementary Table S1).

### QTL Detection in BILs After Different Aging Treatments

**FIGURE 3:**
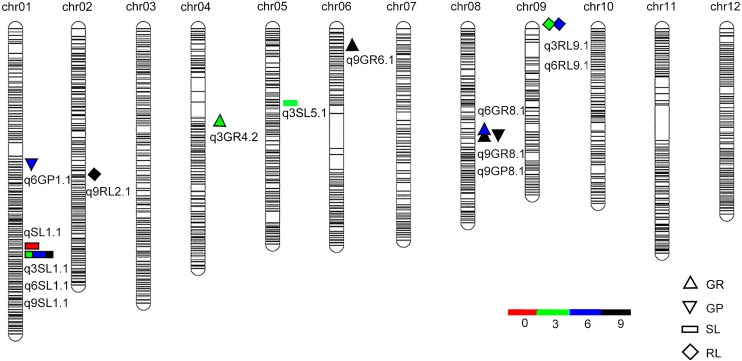
QTLs for seed vigor for artificial aging treatments detected in *Oryza longistaminata* BIL population. QTLs for seed vigor related traits (germination potential, germination rate, seedling length, and root length) under artificial aging treatments. Red, artificial aging for 0 day; Blue, artificial aging for 3 days; Green, artificial aging for 6 days; Dark Black, artificial aging for 9 days.

Totally, 36 QTLs for seed vigor under aging treatments were detected in the BIL population (Table 2 and Supplementary Table S2). Among them, 14 QTLs were harbored by *O. longsiatminata* (Figure 3 and Supplementary Table S2). Of which, apart from seven QTLs were detected in different aging periods (Figure 3), the QTLs *q3SL1.1*, *q6SL1.1*, and *q9SL1.1* were fallen in the same locus flanked by markers Bin1-162 and Bin1-163 on chromosome 1, which explained phenotypic variations ranging from 16.24 to 35.73%, and the relative LOD values ranging from 5.23 to 15.80. Both *q9GP8.1* and *q9GR8.1* were flanked by markers Bin8-127 and Bin8-128 on chromosome 8 with LOD value of 3.04 and 3.05, and explained phenotypic variations of 6.95 and 9.48% in the 9-day treatment, respectively. While *q3RL9.1* and *q6RL9.1* were flanked by markers Bin9-1 and Bin9-2 on chromosome 9, and explain phenotypic variations of 7.5 and 7.66% with LOD value of 2.65 and 2.76 in the 3-day and 6-day aging, respectively.

**Table 2:** QTLs for seed vigor from *O. longistaminata* under artificial aging treatments.

QTLs	Chr.	Position	L-Marker	R-Marker	Interval length (Kb)	LOD	PVE (%)	Add
*q3GR4.2*	4	55 cM	Bin4-54	Bin4-55	146	5.63	14.31	0.03
*q6GR8.1*	8	146 cM	Bin8-118	Bin8-119	80	3.55	9.48	0.03
*q9GR8.1*	8	153 cM	Bin8-127	Bin8-128	90	3.07	9.39	0.04
*q6GP1.1*	1	92 cM	Bin1-83	Bin1-84	90	40.53	114.77	0.39
*q9GP6.1*	6	12 cM	Bin6-2	Bin6-3	118	3.28	7.51	0.05
*q9GP8.1*	8	159 cM	Bin8-127	Bin8-128	90	3.04	6.95	0.04
*qSL1.1*	1	215 cM	Bin1-159	Bin1-160	300	14.96	35.03	1.64
*q3SL1.1*	1	217 cM	Bin1-162	Bin1-163	90	15.80	35.73	1.45
*q3SL5.1*	5	156 cM	Bin5-131	Bin5-132	2000	3.16	6.26	2.35
*q6SL1.1*	1	217 cM	Bin1-162	Bin1-163	90	10.74	28.82	1.11
*q9SL1.1*	1	217 cM	Bin1-162	Bin1-163	90	5.23	16.24	0.86
*q3RL9.1*	9	2 cM	Bin9-1	Bin9-2	520	2.76	7.66	1.02
*q6RL9.1*	9	2 cM	Bin9-1	Bin9-2	520	2.65	7.50	1.03
*q9RL2.1*	2	223 cM	Bin2-247	Bin2-248	300	3.28	7.66	1.68


Additionally, there being 22 QTLs for seed vigor derived from 9311 were identified (Supplementary Figure S3 and Table S2). Of which, except 11 QTLs were scattered on different loci (Supplementary Figure S3), the *qnRL11.1*, *q3RL11.1*, *q6RL11.1*, and *q9RL11.1* four QTLs were mapped at one locus on chromosome 11. Meanwhile, *qnSL4.1* and *q3SL4.1*, *qnGP4.1* and *q3GP4.1*, and *q6GR11.1*, *q6GP11.1*, and *q9GR11.1* three couple of QTLs were mapped together, respectively (Supplementary Figure S3), reflecting one seed vigor trait is usually controlled by locus one regardless aging treatment time.

### Confirmation of the QTLs for High Seed Germinating Rate and Seedling Length

**FIGURE 4:**
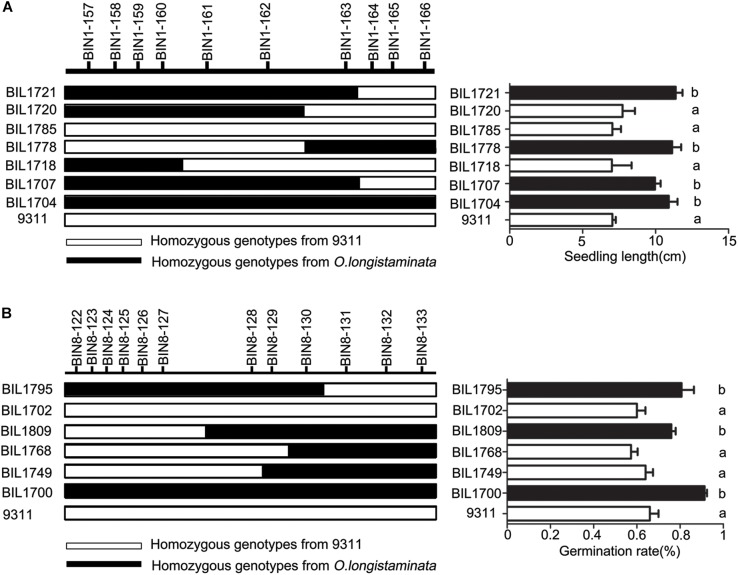
Confirmation of the position of QTLs *q9SL1.1* and *q9GR8.1*. **(A)** Verification of the *q9SL1.1* using 8 BIL lines to delimit it to an interval between molecular marker Bin1-162 and Bin1-163. Black rectangle indicates the homozygous from *O. longistaminata*, white rectangle shows the homozygous from parent 9311, gray indicates the heterozygote genotype. **(B)** Verification of the *q9GR8.1* using 7 BIL lines to delimit it to an interval between molecular marker Bin8-127 and Bin8-128.

In order to confirm the function of genetic locus of *q9SL1.1*, seven BIL lines including BIL 1704, 1707, 1718, 1720, 1721, 1778, and 1785 were selected based on their genotype at *q9SL1.1* (Figure 4A). Among them, BIL 1778, 1721, 1707, and 1704 harbored Bin 1-162∼1-166, Bin 1-157∼1-163, Bin 1-157∼1-163, and Bin 1-157∼1-166 with *O. longistaminata* allele showed higher SL than 9311 (*p* < 0.01). Conversely, at the same region, the BIL lines 1718 carried all alleles from 9311, 1720, and 1785 carried Bin1-157∼1-162 and Bin1-155∼1-162 all exhibited low SL as 9311 regardless of aging treatment or not. In terms of the whole BILs, the lines with *q9SL1.1* shared by *O. longistaminata* exhibited a higher SL than those without *q9SL1.1* no matter in which aging treatment (Supplementary Figure S4A). These results indicate that *q9SL1.1* derived from *O. longistaminata* can significantly increase the SL.

Then, in order to confirm the genetic function of *q9GR8.1* from *O. longistaminta*, six BIL lines including 1700, 1702, 1749, 1768, 1795, and 1809 were further analyzed. Of which, the BIL 1700, 1795, and 1809 carried Bin8-122∼8-133, Bin8-122∼8-130, and Bin8-127∼8-133 all exhibited higher GR than 9311 under 9-day aging treatment (*p* < 0.01). On the contrary, at the same locus, the BIL1702 carried alleles from 9311, 1749, and 1768 harbored Bin8-128∼8-133 and Bin8-129∼8-133 all showed low GR as that of 9311 (Figure 4B). This result reveals that the *q9GR8.1* located between Bin8-127 and Bin8-128, and helps the rice enhancing seed germinating rate under aging treatment. Further, we divided the BIL population into two groups carrying or not of the *q9GR8.1*, to see the genetic effects of *q9GR8.1* on the germinating rate of rice seed. Results showed that the average germinating rate in *q9GR8.1* group including 21 lines was 0.77, significantly higher (*p* < 0.01) than that in another group (131 lines) without *q9GR8.1* when artificially treated for 9 days (Supplementary Figure S4B). By the way, the *q9GR8.1* was in close proximity to *q6GR8.1*, the *q9GR8.1* group also showed higher germinating rate than another group in the 6-day aging treatment (Supplementary Figure S4B). These results were consistent with that *q9GR8.1* was detected at the 6-day and 9-day aging treatment.

## Discussion

In order to understand if the QTLs detected from *O. longistaminta* are new or different from previously reported by others, we compared the currently identified QTLs with that in previous reports by using whole-genome Bin-markers. Out of the 36 QTLs detected in this study, four QTLs including two for germination potential (*qnGP3.1* and *q9GP5.1*), one for seedling length (*q3SL5.1*) and one for root length (*q9RL5.1*) were overlapped with those reported in previous studies. Of which, the QTL *qnGP3.1* is next to *qLTG3-1* which is associated with cold tolerance at germination stage (Challam et al., 2013). Interestingly, previous reports have suggested that some QTLs including those for seed dormancy, germination rate, shoot/root dry weight, as well as some physiological traits such as reducing sugar and total amylase activity (Miura et al., 2002; Zhang et al., 2005) were also mapped to the same locus of *q9GP5.1*, meaning that this locus may be located at a critical cross-point of the cell metabolism, the pathways related to seed germinating potential, seed dormancy, reducing sugar, and total amylase activity are tightly interacted, thus one locus can respond to stress from different adverse environments.

Seed aging or deterioration in rice (*Oryza sativa* L.) is a major problem for agronomic production and germplasm preservation (Lin et al., 2015; Gao et al., 2016; Yan et al., 2018). Kameswara Rao and Jackson (2008) suggested that the seed storability in different rice varieties was: indica>javanica>japonica. However, we know almost nothing about the seed storability of wild rice, and no one QTL for seed-vigor was detected from wild rice. In this study, six novel QTLs for seed germination ability and eight QTLs for seed growth ability under aging treatment were identified from wild rice *O. longsitaminata*. Interestingly, *q9GR8.1* shared same locus with *q9GP8.1* and mapped next to *q6GR8.1*, which contributed to germination capability during aging periods, this locus covered genetic distance about 0.63 cM with physical distance about 90 Kb, it contains eight predictable protein-encoding genes (Table 3). Meanwhile, *q9SL1.1* co-located with *q3SL1.1* and *q6SL1.1*, which contributes to seedling length during all the artificial aging treatment. The *q3SL1.1*, *q6SL1.1*, and *q9SL1.1* each explains 35.73, 28.82, and 16.24% of the phenotypic variation, meaning that this locus most possibly a critical region for seed vigor in *O. longsitaminata*. Importantly, this locus covers about 1.63 cM corresponding to about 90 Kb physical distance, contains only 14 predictable protein-encoding genes (Table 3). This will establish robust foundation for further cloning the gene for seedling length against seed aging.

**Table 3:** The predicted functional genes at locus of *q9GR8.1* and *q9SL1*.*1.*

QTL	Locus names	Cds coordinates	Gene function
*q9GR8.1*	MH08t0020600-01	766239–767089	Hypothetical protein
	MH08t0020700-01	770084–776883	Splicing regulatory glutamine/lysine-rich protein 1
	MH08t0020800-01	781803–783479	60S acidic ribosomal protein P1
	MH08t0020900-01	784159–812160	Putative vacuolar protein sorting-associated protein 13E
	MH08t0021000-02	813697-816322	Exosome complex component MTR3
	MH08t0021100-01	818467–822202	Probable beta-1; 3-galactosyltransferase 6
	MH08t0021200-01	825763–830467	Kinesin-1-like protein PSS1
	MH08t0021300-01	36523094–36523616	28 kDa ribonucleoprotein; chloroplastic
*q9SL1.1*	MH01t0725700-03	36437075–36439562	Amino acid permease 8
	MH01t0725800-01	36452273–36456857	Amino acid permease 5
	MH01t0725900-01	36458041–36462232	4, 5-DOPA dioxygenase extradiol-like protein
	MH01t0726000-01	36463217–36464789	4; 5-DOPA dioxygenase extradiol-like protein
	MH01t0726100-01	36465532–36466669	Hypothetical protein
	MH01t0726200-01	36467056–36469230	Putative AC transposase
	MH01t072630001	36470755–36471870	Hypothetical protein OsI_04655
	MH01t0726400-01	36473946–36474632	hypothetical protein OsI_04656
	MH01t0726500-02	36476633–36479730	Beta-glucanase-like protein
	MH01t0726600-01	36478797–36482441	Ribosomal N-lysine methyltransferase 3
	MH01t0726700-01	36486749–36490615	hypothetical protein OsI_04659
	MH01t0726800-01	36495623–36496104	hypothetical protein OsI_04660
	MH01t0726900-01	36500895–36503363	Retrotransposable element Tf2 155 kDa protein type 1
	MH01t0727000-01	36523094–36523616	Hypothetical protein
	MH01t0727100-01	36528435–36534828	UDP-glucuronate:xylan alpha-glucuronosyltransferase 1


Backcross inbred lines from the wild rice is a powerful tool to improve the rice breeding. In this study, 14 novel seed vigor QTLs from *O. longistaminata* through a BC_2_F_20_ BIL population were identified, further validate the importance of *O. longistaminata* to rice improvement. As release of high-quality genome of *O. longistaminata*, combined with construction of near isogenic lines, decryption of the secret of seed vigor of *O. longistaminata* will be accelerated, and which will greatly help the utilization of *O. longistaminata* in rice breeding programs.

## Author Contributions

SL and WL conceived and planned the work. JJ, XL, LW, WX, and GP performed phenotypic screening. SL, NL, and JJ developed the population. JJ and WL analyzed the genotypic data. JJ, WL, and SL drafted the manuscript.

## Conflict of Interest Statement

The authors declare that the research was conducted in the absence of any commercial or financial relationships that could be construed as a potential conflict of interest.

## Footnotes

1 https://www.rdocumentation.org/packages/corrplot/versions/0.84
