# Supplementary material for: QTL Mapping of Seed Vigor of Backcross Inbred Lines Derived From Oryza longistaminata Under Artificial Aging
Source: Front Plant Sci. 2018 Dec 21;9:1909. doi: 10.3389/fpls.2018.01909 (PMC6308630; doi:10.3389/fpls.2018.01909)
Supplement: Supplementary file 1 [file Data_Sheet_1.docx]

**Supplementary Figures and Tables**

**
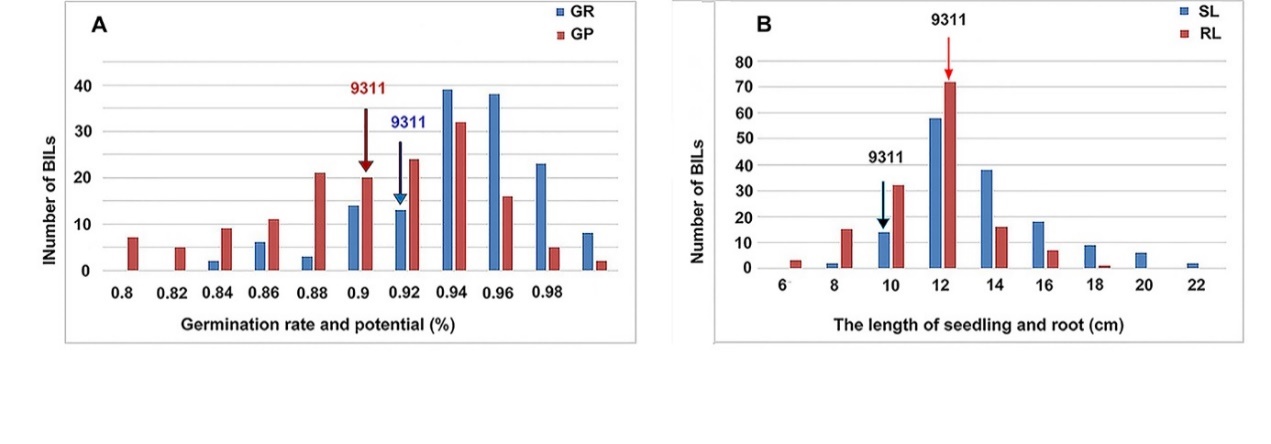
**

**Supplementary Fig. 1 Distribution frequency of the seed vigor trait of BIL population under normal treatment.** A indicates the germination potential and germination rate, and B shows the seedling length and root length.


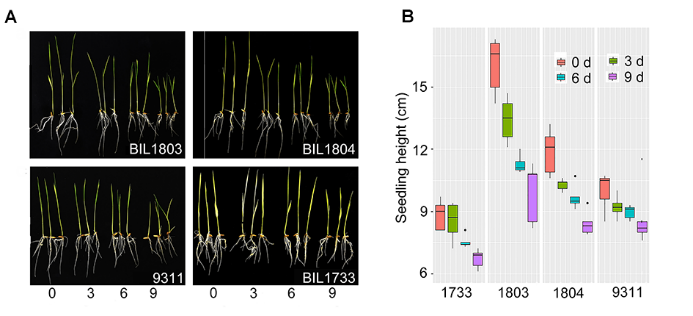


**Supplementary Fig. 2 The plants stature of 9311 and three BIL lines under artificial aging treatment.** A, the seedling plants; B, the statistical box plot of the seedling length, red: control; blue: treatment 3 days; green: treatment 6 days; purple: treatment 9 days.


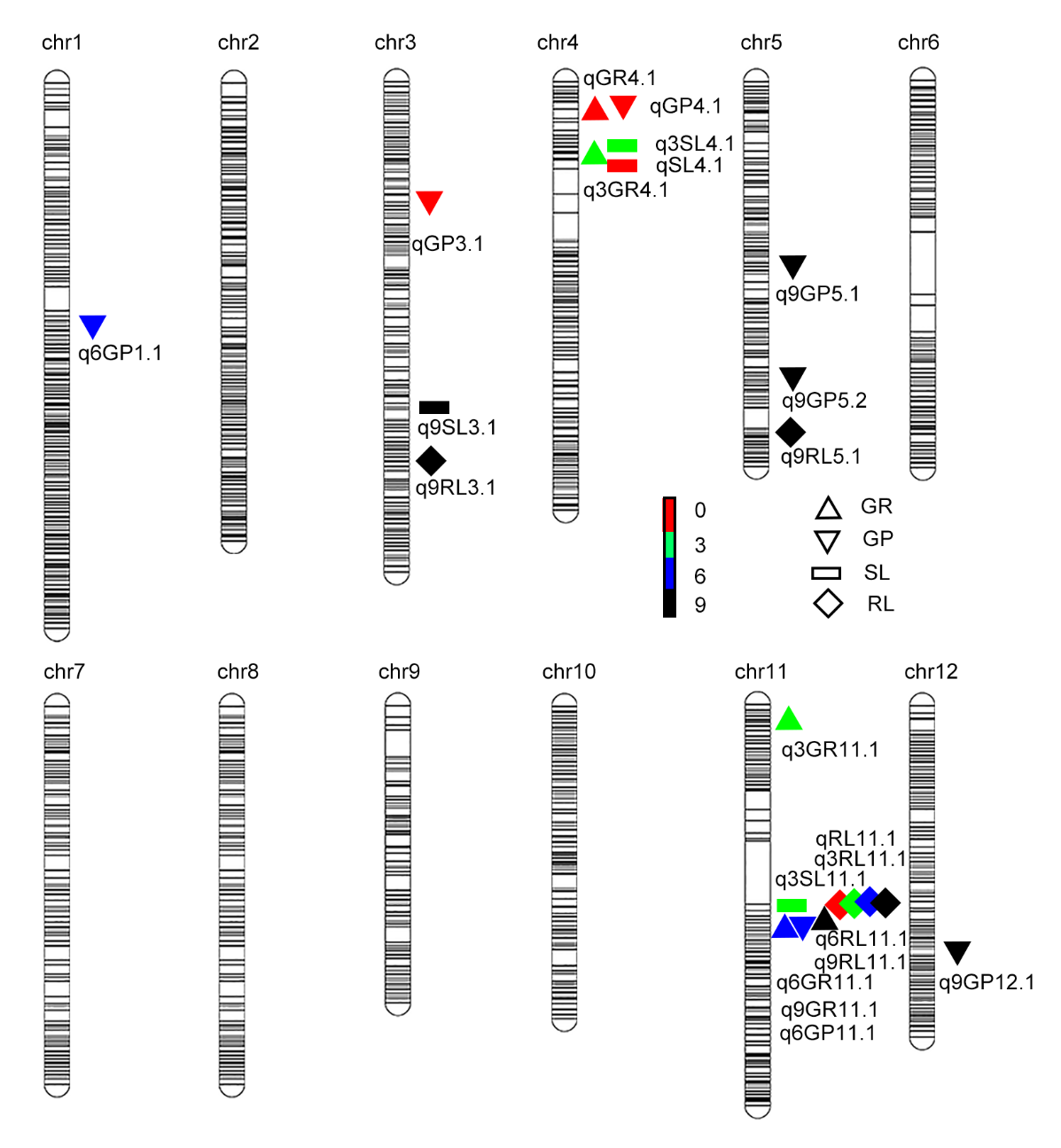


**Supplementary Fig. 3** **QTLs for seed vigor for artificial aging treatments detected in BIL population shared by 9311**. QTLs for seed vigor related traits (germination potential, germination rate, seedling length and root length) under artificial aging treatments. Red, artificial aging for 0 day; blue, artificial aging for 3 days; green, artificial aging for 6 days; dark black, artificial aging for 9 days. Positive triangle, germination rate (GR); inverted triangle, germination potential (GP); rectangle, seedling length (SL); diamond, root length (RL).


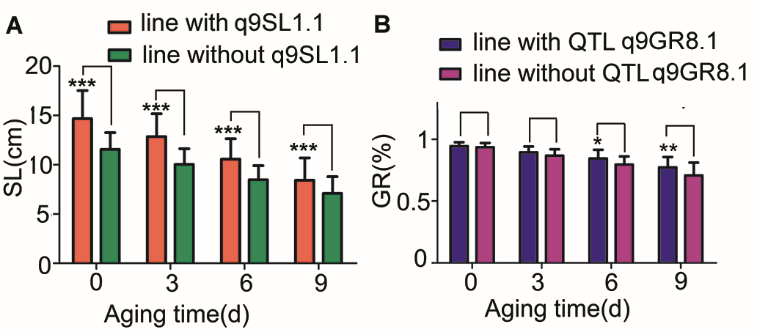


**Supplementary Fig. 4 QTLs *q9SL1.1* and *q9GR8.1* from *O. longistaminata* contributed to seed-vigor related traits under artificial aging** A. with the QTL *q9SL1.1* BILs show significance high seedling length than the BILs which not shared with the QTL *q9SL1.1* with four artificial aging treatments. B. when under 0 and 3 days aging condition, there is no significance difference between the BILs with or without the QTL *q9GR8.1*, but under 6 and 9 days aging condition, with *q9GR8.1* BILs performance better than BILs without *q9GR9.1*. Significance levels: *, P < 0.05; **, P < 0.01 and ***, P < 0.0001.

**Supplementary Table 1. Correlation analysis of the four seed vigor related traits**.

| trait | treatments | GP | GR | SL | RL |
| --- | --- | --- | --- | --- | --- |
| GP | 0 |  |  |  |  |
|  | 3d aging |  |  |  |  |
|  | 6d aging |  |  |  |  |
|  | 9d aging |  |  |  |  |
| GR | 0 | 0.89****** |  |  |  |
|  | 3d aging | 0.69** |  |  |  |
|  | 6d aging | 0.9** |  |  |  |
|  | 9d aging | 0.96** |  |  |  |
| SL | 0 | -0.09 | -0.05 |  |  |
|  | 3d aging | 0.13 | 0.17* |  |  |
|  | 6d aging | 0.03 | 0.02 |  |  |
|  | 9d aging | -0.03 | -0.01 |  |  |
| RL | 0 | 0.02 | 0.03 | 0.22** |  |
|  | 3d aging | 0.04 | -0.04 | 0.09 |  |
|  | 6d aging | -0.05 | -0.05 | 0.27** |  |
|  | 9d aging | 0.03 | 0.03 | 0.45** |  |

Significance levels: * P < 0.05, and ** P < 0.01. Lower diagonal present the same treatment wise correlations. Person method was carried out to calculate the correlation in this study.

**Supplementary Table 2. Putative QTLs for seed vigor in the BILs under aging stress derived from 9311.**

| ***QTLs*** | **Chromosome** | **Position** | **Left Marker** | **Right Marker** | **LOD** | **PVE (%)** | **Add** |
| --- | --- | --- | --- | --- | --- | --- | --- |
| *qnGR4.1* | 4 | 20cM | BIN4-13 | BIN4-14 | 3.3813 | 10.9278 | -0.0147 |
| *q3GR4.1* | 4 | 37cM | BIN4-35 | BIN4-36 | 9.9432 | 27.2070 | -0.0364 |
| *q3GR11.1* | 11 | 58cM | BIN11-48 | BIN11-49 | 2.9990 | 7.7378 | -0.0148 |
| *q6GR11.1* | 11 | 304cM | BIN11-135 | BIN11-136 | 3.9316 | 10.4119 | -0.0584 |
| *q9GR11.1* | 11 | 302cM | BIN11-134 | BIN11-135 | 4.7531 | 13.2605 | -0.1162 |
| *qnGP3.1* | 3 | 156cM | BIN3-75 | BIN3-76 | 2.7947 | 15.7109 | -0.0622 |
| *qnGP4.1* | 4 | 19cM | BIN4-13 | BIN4-14 | 2.9899 | 9.3253 | -0.0204 |
| *q6GP1.2* | 1 | 94cM | BIN1-85 | BIN1-86 | 49.8463 | 171.4421 | -0.4177 |
| *q6GP11.1* | 11 | 303cM | BIN11-135 | BIN11-136 | 4.1736 | 6.6499 | -0.0759 |
| *q6GP12.1* | 12 | 176cM | BIN12-182 | BIN12-183 | 3.3086 | 5.9074 | -0.0290 |
| *q9GP5.1* | 5 | 159cM | BIN5-134 | BIN5-135 | 3.8301 | 8.9926 | -0.2058 |
| *q9GP5.2* | 5 | 309cM | BIN5-205 | BIN5-206 | 3.0648 | 7.0671 | -0.0375 |
| *qnSL4.1* | 4 | 56cM | BIN4-55 | BIN4-56 | 3.9157 | 7.6364 | -1.1777 |
| *q3SL4.1* | 4 | 42cM | BIN4-42 | BIN4-43 | 2.6127 | 4.7710 | -0.6947 |
| *q6SL11.1* | 11 | 145cM | BIN11-102 | BIN11-103 | 2.9635 | 6.9983 | -0.6992 |
| *q9SL3.1* | 3 | 332cM | BIN3-178 | BIN3-179 | 3.5878 | 10.94 | -1.1694 |
| *qnRL11.1* | 11 | 145cM | BIN11-102 | BIN11-103 | 3.5361 | 10.8258 | -0.9114 |
| *q3RL11.1* | 11 | 145cM | BIN11-102 | BIN11-103 | 3.2248 | 8.9712 | -0.7688 |
| *q6RL11.1* | 11 | 136cM | BIN11-100 | BIN11-101 | 3.9298 | 17.6261 | -1.0396 |
| *q9RL3.1* | 3 | 352cM | BIN3-213 | BIN3-214 | 3.1279 | 7.1639 | -0.8839 |
| *q9RL5.1* | 5 | 258cM | BIN5-184 | BIN5-185 | 4.0736 | 9.3032 | -0.8259 |
| *q9RL11.1* | 11 | 147cM | BIN11-102 | BIN11-103 | 5.6786 | 14.2183 | -0.8473 |

Notes: RL, represents root length; SL, represents seedling length; GP, represents germinating potential; GR, represents germinating rate.
